# *Staphylococcus aureus* Co-Infection in COVID-19 Patients: Virulence Genes and Their Influence on Respiratory Epithelial Cells in Light of Risk of Severe Secondary Infection

**DOI:** 10.3390/ijms251810050

**Published:** 2024-09-18

**Authors:** Lidia Piechowicz, Katarzyna Kosznik-Kwaśnicka, Tomasz Jarzembowski, Agnieszka Daca, Agnieszka Necel, Ada Bonawenturczak, Olesia Werbowy, Małgorzata Stasiłojć, Anna Pałubicka

**Affiliations:** 1 Department of Medical Microbiology, Faculty of Medicine, Medical University of Gdansk, Debowa 25, 80-204 Gdansk, Poland; tomasz.jarzembowski@gumed.edu.pl (T.J.); agnieszka.necel@gumed.edu.pl (A.N.); 2 Department of Physiopathology, Medical University of Gdansk, Debinki 7, 80-211 Gdansk, Poland; agnieszka.daca@gumed.edu.pl; 3 Department of Microbiology, Faculty of Biology, University of Gdansk, Wita Stwosza 59, 80-308 Gdansk, Poland; a.bonawenturczak.074@studms.ug.edu.pl (A.B.); olesia.werbowy@ug.edu.pl (O.W.); 4 Department of Cell Biology and Immunology, Intercollegiate Faculty of Biotechnology of University of Gdansk and Medical University of Gdansk, Debinki 1, 80-211 Gdansk, Poland; malgorzata.stasilojc@gumed.edu.pl; 5 Specialist Hospital in Koscierzyna Sp. z o.o., Department of Laboratory and Microbiological Diagnostics, Koscierzyna, Alojzego Piechowskiego 36, 83-400 Koscierzyna, Poland; apalubicka@op.pl

**Keywords:** *Staphylococcus aureus*, COVID-19, SARS-CoV-2, adhesion

## Abstract

Pandemics from viral respiratory tract infections in the 20th and early 21st centuries were associated with high mortality, which was not always caused by a primary viral infection. It has been observed that severe course of infection, complications and mortality were often the result of co-infection with other pathogens, especially *Staphylococcus aureus*. During the COVID-19 pandemic, it was also noticed that patients infected with *S. aureus* had a significantly higher mortality rate (61.7%) compared to patients infected with SARS-CoV-2 alone. Our previous studies have shown that *S. aureus* strains isolated from patients with COVID-19 had a different protein profile than the strains in non-COVID-19 patients. Therefore, this study aims to analyze *S. aureus* strains isolated from COVID-19 patients in terms of their pathogenicity by analyzing their virulence genes, adhesion, cytotoxicity and penetration to the human pulmonary epithelial cell line A549. We have observed that half of the tested *S. aureus* strains isolated from patients with COVID-19 had a necrotizing effect on the A549 cells. The strains also showed greater variability in terms of their adhesion to the human cells than their non-COVID-19 counterparts.

## 1. Introduction

Pandemics from viral respiratory tract infections in the 20th and early 21st centuries were associated with high mortality, which was not always caused by a primary viral infection. It has been observed that the severe course of these infections, complications and mortality were often the result of co-infection with other pathogens, one of them being *Staphylococcus aureus* (*S. aureus*) [1,2,3,4,5]. In the case of the COVID-19 pandemic caused by the SARS-CoV-2 virus, a similar trend has been observed. It was shown that among the bacteria isolated from the lower respiratory tracts of patients with COVID-19, artificially ventilated due to acute respiratory distress syndrome, as many as 70% were *S. aureus* strains [6]. In addition, patients with COVID-19infected with *S. aureus* had a particularly high mortality rate (61.7%) that was significantly higher compared to patients infected with viral SARS-CoV-2 only [7].

Taking the above into account, undoubtedly, the key issue in the diagnostic and therapeutic management of patients with COVID-19 is the risk of bacterial co-infection in the respiratory tract from *S. aureus* strains, which may lead to an intensification of infection symptoms and sometimes death.

Asymptomatic colonization (carrier) in adults by *S. aureus* amounts to 30% to 50% and most often occurs in the nasal vestibule and upper respiratory tract [8,9]. Among them, 20–35% are classified as permanent carriers, while the remaining 30–70% of the population acts as intermittent carriers [8]. It is known that carrying *S. aureus* strains in the upper respiratory tract is a risk factor for subsequent invasive infection in patients, as well as the transmission of infection between patients. Many cases of staphylococcal bacteremia have an endogenous origin, originating in the mucous membrane of the nasal vestibule [10].

*S. aureus* can asymptomatically colonize the respiratory tract of healthy people, as well as cause infections of varying severity from local, non-invasive and relatively mild skin infections to severe, life-threatening sepsis, infective endocarditis and necrotizing pneumonia [11,12]. The diversity of infections associated with *S. aureus* is the result of the production of multiple virulence factors and the adaptation of strains to different host environments. Adaptation allows bacteria to colonize the host organism, defend against the immune system, spread into the infected organism and destroy its cells and tissues [13].

To start an infection, *S. aureus* uses various proteins (adhesins) associated with the cell surface that facilitate the attachment of bacteria to the host tissue and its destruction [14]. The most important adhesins of staphylococci colonizing the respiratory tract include fibronectin-binding (FnBP) and fibrinogen-binding (Clf) adhesins [8,14]. It has been proven that FnBPs mediate the adhesion of *S. aureus* to human respiratory epithelial cells, endothelial cells and fibroblasts where the internalization process can occur [15,16]. In addition, staphylococci with *fnbA* and *fnbB* genes are often isolated from invasive infections such as endocarditis and arthritis [17]. After adhesion and internalization, bacteria can grow and activate the production of toxins that damage and destroy host cells, largely controlled by quorum-sensing systems [18,19,20]. During the infection process, toxins allow bacteria to penetrate and destroy deep tissue structures, obtain nutrients for their growth and defend against the immune system [20]. Undoubtedly, pyrogenic exotoxins and invasins, such as toxic shock toxin (TSST-1), enterotoxins, exfoliatins (ETs) or Panton–Valentine leukocidin (PVL), play a key role in the pathogenesis of acute staphylococcal infections [21,22]. PVL, in addition to purulent local lesions, is responsible for staphylococcal necrotizing pneumonia [23]. The severity of symptoms of the SARS-CoV-2 infection may be related to the production by *S. aureus* of superantigens such as toxic shock toxin (TSST-1), staphylococcal enterotoxins A-E (SEA-SEE) or exfoliative toxins (ETs). Superantigens cause a non-specific polyclonal activation of T lymphocytes, leading to symptoms of toxic shock and even death in the patient [24]. After the acute phase of infection, *S. aureus* bacteria strive to survive in the host, avoiding recognition by the immune system [25,26,27].

Our previous studies have shown that the *S. aureus* strains isolated from patients with COVID-19 had a different protein profile than strains in non-COVID-19 patients [28]. Therefore, the study aims to investigate the *S. aureus* strains isolated from patients with COVID-19, in terms of their pathogenic potential, by analyzing their adhesion, cytotoxicity and penetration into human cell lines derived from the patients’ respiratory tract and detecting selected genes of toxins and staphylococcal adhesins.

## 2. Results

### 2.1. The Presence of Toxin and Adhesin Genes

The presence of toxin and adhesin genes was assessed with PCR-based techniques using previously established protocols [29,30,31]. All of the *S. aureus* COVID-19 strains harbored the fibronectin-binding protein B gene (*fnbB*), and almost all strains, except for five (no. 2, 4, 8, 12 and 17), possessed the *fnbA* gene (80.8%) (Table 1 and Table S3). As opposed to the COVID-19 strains, only seven of the non-COVID-19 *S. aureus* strains (7/21, 33.3%) were positive for the *fnbB* gene (*p* < 0.01), but all strains were positive for the *fnbA* gene (100%). The occurrence of toxin genes among the 26 analyzed COVID-19 *S. aureus* strains was low (3/26, 11.5%), while the group of non-COVID-19 strains was higher (6/21, 28.7%; *p* > 0.05). It was observed that in the COVID-19 strain group, only the *sec* and *tst* toxin genes were detected, while in the non-COVID-19 strains, other toxin genes were detected, such as *sea*, *seb* and *pvl*, with the most frequent being the *sea* gene (*p* < 0.05).

### 2.2. The Adhesion of S. aureus Strains to the Pulmonary Epithelial Cells (A549 Line)

The ability of bacteria to adhere to host cells can be the primary step in the pathogenesis of diseases [11]. The adhesion ability of COVID-19 and non-COVID-19 *S. aureus* to the pulmonary epithelial cells (A549 line) was assessed using flow cytometry.

**Figure 1:**
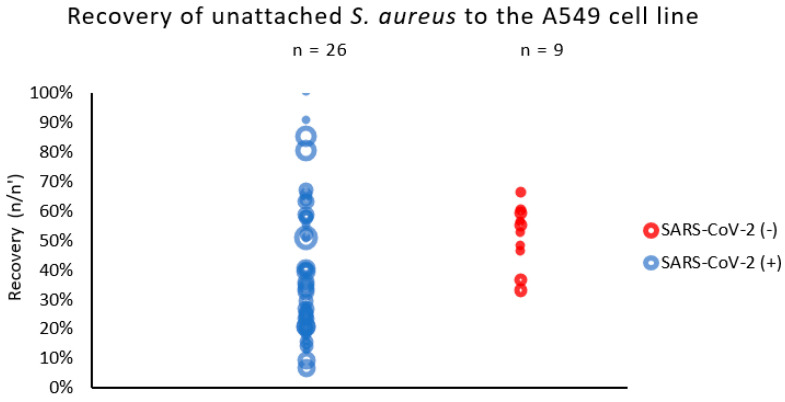
Adhesion of S. aureus strains measured by % of recovered bacteria not adhered to respiratory epithelial cells (A549 line). n/n′—ratio of counts per 60 s of sample flow before and after exposition to epithelial cells (A549). The circle represents the result (median) of a single strain; the size of the circle represents the relative initial inoculum of the sample.

We observed that the *S. aureus* strains isolated from patients with COVID-19 (SARS-CoV-2 (+)) were more diverse in terms of their adhesion to the respiratory epithelial cells than the strains derived from non-COVID-19 patients (Figure 1). The recovery of unattached *S. aureus* strains to the A549 cell line varied from 7% to 100% in patients with SARS-CoV-2. In the non-COVID-19 group (SARS-CoV-2 (−)), the diversity of unattached *S. aureus* to the A549 was definitely lower and varied from 66% to 33% only.

**Figure 2:**
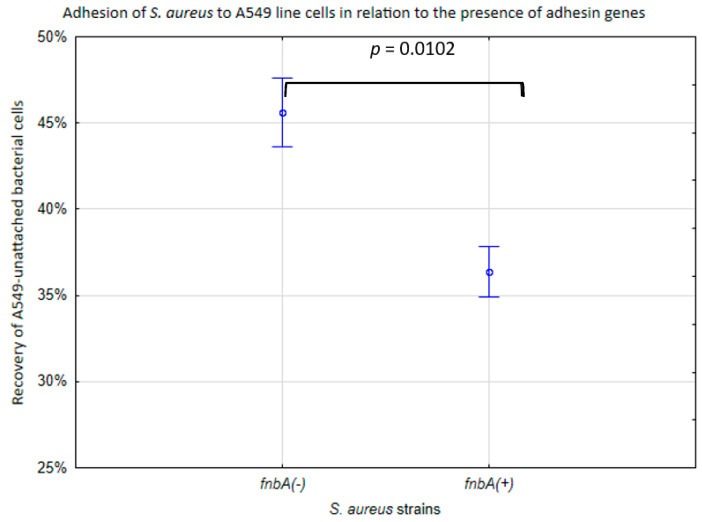
Adhesion of the *S. aureus* cell line in relation to the presence of adhesin genes. % of recovery of unattached A549 bacterial cells to the initial bacterial suspension. The vertical bars represent standard errors. The statistical analysis was performed using the Kruskal–Wallis test.

The variation in adhesion to A549 cells in SARS-CoV-2 (+) *S. aureus* was associated with the *fnbp* genes. In the *fnbA*-deficient strains (*fnbA* (−)), the median recovery of unattached bacteria was 46% of the bacterial cells, while in the *fnbA* (+) strains, this parameter was lower (36%) (*p* < 0.05) (Figure 2).

### 2.3. The Penetration of S. aureus Strains into the Pulmonary Epithelial Cells

**Figure 3:**
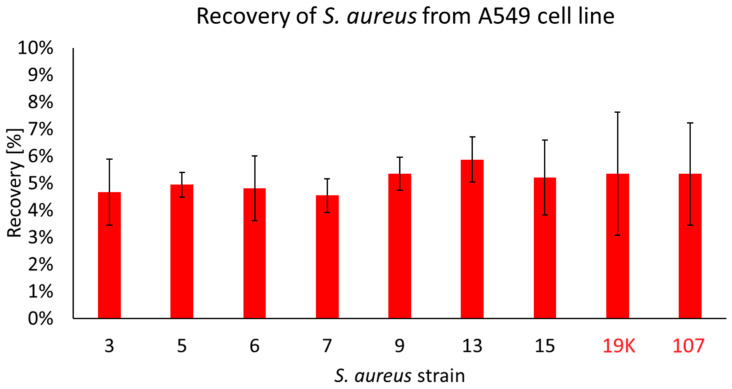
The percentage of COVID-19 (black numbers) and non-COVID-19 (red numbers) *S. aureus* cells recovered from the inside of A549 pulmonary epithelial cells. The arithmetic mean of the triplicates, including the error bars that represents the SD. The statistical analysis was performed using a *t*-test. No statistical differences were found between the strains recovered from the inside of A549 cells.

*S. aureus* is able to invade host cells and is capable of intracellular survival for extended periods of time while regulating its metabolism and toxin expression [24]. During our studies, we have observed that only seven strains coming from the patients with COVID-19 (all possessing *fnbA* and *fnbB* genes) were recovered from the inside of the A549 cells, as follows: no. 3, 5, 6, 7, 9, 13 and 15. In the non-COVID strains, two of the tested strains were discovered to be able to migrate to the inside of the cell: 19K and 107. The CFU/mL of the recovered strains was, on average, 5 × 10^5^ CFU/mL and accounted for ~6% of the initial infective dose. No statistically significant differences were observed between the strains (Figure 3).

### 2.4. The Effect of S. aureus Strains on the Viability of Respiratory Epithelial Cells (A549 Line) and Their Membrane Integrity

*S. aureus* has a negative impact on the survival of host cells as a result of the massive production of various toxins [32]. To determine whether the *S. aureus* strains from the COVID-19 and non-COVID-19 patients had cytotoxic activity, a line of A549 respiratory epithelial cells was inoculated with the tested strains for 4 h and the cytotoxicity was measured using neutral red staining (viability) and LDH release levels. Afterwards, we compared the values of the COVID-19 strains, non-COVID-19 strains, the A549 cell line without bacteria (negative control) and the A549-lysed cells (positive control).

**Figure 4:**
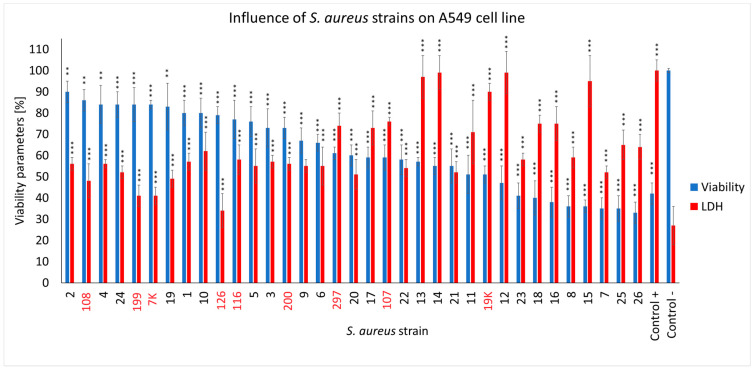
The cell viability and LDH release assay for the A549 cells infected with different *S. aureus* strains (COVID-19 strains are marked in black, and non-COVID-19 strains are marked in red) after 4 h of incubation compared to the uninfected cells and the lysis-buffer-treated positive control. The mean values from the three independent experiments are shown, with the error bars representing the SD. The statistical analysis was performed using the Kruskal–Wallis test, followed by Dunn’s multiple comparison test for the values with a nonparametric distribution, with *** *p* < 0.001 and ** *p* < 0.01.

We observed that the *S. aureus* COVID-19 strains differed in their cytotoxic effects in the cells from the A549 cell line (Figure 4). As many as 16 strains out of the 26 examined (61.5%) caused a particular decrease in the viability of the lung epithelial cells in the range of ~32–60%, with half of them causing an additional evident decrease in the membrane integrity of the human cells up to 30–40% (strain no. 7, 8, 15, 16, 18, 23, 25 and 26). The remaining COVID-19 strains (38.5%) showed a lower cytotoxicity in the A549 cells, leaving ~65–88% of them alive (strain no. 1, 2, 3, 4, 5, 6, 9, 10, 19 and 24). Compared to the COVID-19 strains, the non-COVID-19 strains induced less of a decline in cell health, leaving ~70% to 85% of the human cells alive (strain no. 7K, 108, 116, 126, 199, 200). Infection in the strain no. 297, 107 and 19K had a visible necrotic effect on the cells (LDH levels exceeded the viability parameters). The A549 cell count present within the4 h was only ~60%. Both the *S. aureus* COVID-19 and non-COVID-19 strains compromised the membrane integrity of the A549 cells, with the first group of strains acting more effectively. As for the effect of the COVID-19 strains on the human cells, the LDH release levels were mostly higher compared to the non-COVID-19 strains (~90–50% vs. ~72–41%) (Figure 4).

## 3. Discussion

The SARS-CoV-2 virus can cause serious epidemics, resulting in high morbidity and mortality. Epidemiological data suggest that bacterial co-infections are the main cause of an increase in deaths. In this context, *S. aureus* occurs as a common etiological agent of infections. *S. aureus* is a pathogen that colonizes the nasopharyngeal area of many healthy individuals [5,6]. Several studies have reported an association between colonization and invasive infections, such as pneumonia and bloodstream infections leading to sepsis or septic shock, particularly in hospitalized patients or patients with concomitant viral infections [10]. Many of these are associated with particularly high mortality rates [7,8].

In this study, we investigated *S. aureus* strains isolated from the nasal vestibule of patients with COVID-19 in terms of its pathogenic potential in light of the risk of a severe secondary infection. We analyzed the adhesion, cytotoxicity and penetration into the human cell line of *S. aureus* COVID-19 strains derived from the respiratory tracts of patients and detected the selected genes of staphylococcal toxins/adhesins.

*S. aureus* presents a wide range of pathogenic factors that enable this bacterium to colonize the host organism, defend against the immune system, spread into the infected body and destroy cells and tissues. To cause infection, *S. aureus* produces specific virulence factors, such as adhesins and toxins, that work together to destroy the host tissues and develop immune insensitivity [14,33,34].

To initiate the infection, *S. aureus* needs to attach to the host tissue and invade the host cells; and surface proteins of bacteria such as MSCRAMM (microbial surface components recognizing adhesive matrix molecules) play a key role in the adhesion phase, host cell invasion and immune response evasion [8,14]. It has been proven that some of these molecules, such as FnBPs, mediate adhesion to the human respiratory tract epithelial cells, endothelial cells and fibroblasts, where bacterial internalization can also occur [35].

In this study, the adhesion of *S. aureus* strains from patients with COVID-19 to human lung endothelial cells (A549 line) was analyzed. The results obtained via flow cytometry showed more diverse adhesion among these strains compared to staphylococci isolated from infections (pneumonia, sepsis) in non-COVID-19 patients. In addition, among the COVID-19 strains, *S. aureus*, with genes of both adhesins (FnBPA, FnBPB) showed greater adhesion capacity compared to strains without FnBPA. Our results differ from those described by Peacock et al., in which the number of *fnb* genes did not influence overall fibronectin binding, but the isolates associated with invasive diseases (endocarditis or primary septic arthritis and/or osteomyelitis) were more likely to have two adhesin genes [17]. In most of the COVID-19 strains we studied, we detected genes that encode the production of FnBPA and FnBPB adhesins, increasing the risk of more severe symptoms during infection.

In the next steps of the work, we examined how infection of the cell culture (A549 line) influences cell viability and cell membrane integrity. *S. aureus* can induce host cell death through programmed forms of cell death, such as pyroptosis, apoptosis, necroptosis and autophagy [36,37,38,39]. During this process, *S. aureus* secretes toxins and exoenzymes that can trigger tissue destruction and make it easier for bacteria to spread [40]. We found that more than 60% of the tested COVID-19 strains had a necrotic effect on the pulmonary epithelial cells (A549 line) because low cell survival and high lactate dehydrogenase (LDH) levels were achieved. In addition, we observed that both the COVID-19 and non-COVID-19 *S. aureus* strains, isolated from patients who were infected, compromised the membrane integrity of the A549 cells, with the first group of strains acting more effectively.

To analyze whether there was a correlation between cytotoxicity and staphylococcal toxin genes, we reviewed the presence of *S. aureus* exotoxin genes, such as Panton–Valentine leucocidin (PVL), exfoliative toxins (ETA and ETB), toxic shock syndrome toxin-1 (TSST-1) and classic enterotoxins (SEA-SED). The choice was dictated by the possible participation of specific toxins in the pathogenesis of invasive infections such as pneumonia (e.g., PVL) or sepsis and septic shock (e.g., TSST-1, enterotoxins, exfoliative toxins). By the genotypic analysis of toxin genes, we found only a few genes in both the COVID-19 and non-COVID-19 *S. aureus* groups. A low frequency of *tst* and *sec* genes were detected (7.7% and 3.4%) among the COVID-19 strains. The enterotoxin genes *sea*, *sec* and *pvl* were found in the non-COVID-19 strains, with a frequency of 19% (*p* < 0.05), 4.8% and 4.8%, respectively. TSST-1 and SEC are superantigens produced by *S. aureus* that can activate CD4 T cells in a predominantly nonspecific manner to produce large amounts of cytokines and lead to a systemic inflammatory response [38]. In addition to influencing the development of staphylococcal food poisoning, enterotoxins affect the Th1, Th2 and Th22 cells, inducing apoptosis. Additionally, the SEC toxin can lead to Alzheimer’s disease and cancer [39,40]. A higher prevalence of toxin genes has been reported by other authors [41,42]. According to research conducted by Von Eiff et al. [10], the frequency of toxin genes in *S. aureus* isolated from blood and nasal swabs were 20.3% (*tst)* and 11.2% (*sec*). No significant difference in gene presence was observed between the blood isolates and those isolates derived from nasal swabs [41]. The high toxicity and low frequency of genes in the selected toxins from the COVID-19 *S. aureus* strains we studied do not correlate with previous reports of a positive relationship between cytotoxicity and toxin production [37].

After the tissues are destroyed and the bacteria spread, *S. aureus* attacks the host cell to settle the infection. One way to evade the immune system is to take refuge in host cells. In addition to phagocytes such as neutrophils and monocytes [43], this has also been demonstrated in a number of non-phagocytic cells, including epithelial and endothelial cells, keratinocytes and osteoblasts. Invasion of non-phagocytic cells contributes to the chronicity of infection and is mediated in part by FnBPs [41,42]. FnBPs bind fibronectin on the cell surface via a tandem-β-zipper mechanism, and after internalization, *S. aureus* escapes from the phagosome [43,44]. Therefore, we investigated the invasiveness of staphylococcal isolates by infecting the pulmonary epithelial cells (A549 line) and measuring the number of intracellular bacteria. In the last decade, *S. aureus* has been increasingly perceived as an intracellular pathogen [45]. *S. aureus* is able to invade host cells and is capable of intracellular survival for extended periods of time while regulating its metabolism and toxin expression [25]. We have demonstrated that host cell penetration is not a characteristic feature of the *S. aureus* strains isolated from the nasal vestibule of COVID-19 patients. Only 7 out of 26 of the tested strains were recovered from the inside of A549 cells, and the CFU/mL of the recovered strains were, on average, 5 × 10^5^ CFU/mL, accounting for ~6% of the initial infective dose. This means that most of the tested COVID-19 *S. aureus* strains do not have the ability to penetrate epithelial cells and cause a chronic infection. We have observed that all seven strains isolated from the inside of A549 cells carried genes encoding both FnBPs (A and B), increasing the likelihood of the intra-cellular penetration of bacteria.

*S. aureus* can also make use of the primary harms resulting from other pathogens or predisposing conditions, for example, in pulmonary infections that have been initiated by a viral infection in an opportunistic fashion [46,47]. It has been noted that the SARS-CoV-2 infection can damage the cells and the lungs’ infrastructure [48]. Subsequently, the changed condition enables bacteria to increase their adherence and ease of invasion [49]. Bacterial co-infections, defined as the diagnosis at the time of, or within 24 h of, COVID-19 hospital admission, affect only a few percent of patients [50,51,52]. However, the contribution of *S. aureus* to bloodstream and respiratory infection in such patients is high and, according to various authors, amount to 31% [53] and over 50% [50].

For this study, we have collected *S. aureus* strains colonizing patients with the SARS-CoV-2 virus infection. The tested strains turned out to have different adhesive properties, a high cytotoxicity in the respiratory epithelium cells and almost all of them had two main adhesins (FnBPA and FnBPB) associated with invasive diseases [14]. It can, therefore, be assumed that the tested *S. aureus* strains have the potential to cause infection, and are all the more likely when the primary virus infection facilitates a secondary bacterial infection, even in low virulent strains [54].

Our study has limitations. First, we detected only some genes encoding virulence factors; however, *S. aureus* can produce a lot of different factors responsible for its virulence. Due to our small sample size, we cannot exclude the possibility that other virulence factors are also involved in pulmonary epithelial cell destruction. Second, the number of tested strains was quite small, which resulted from the use of samples obtained from one hospital, the short period of time for sample collection and the low frequency of *S. aureus* strains isolated from them. Therefore, it is recommended that future studies should also take into account the above points.

In conclusion, our results show that the *S. aureus* strains isolated from patients with COVID-19 exhibit a high efficacy in their destructive impact on pulmonary epithelial cells; therefore, the *S. aureus* strains we studied possess properties that pose a risk serious infections developing in patients with COVID-19.

## 4. Materials and Methods

### 4.1. Strain Collection and Identification

Between December 2021 and February 2022, 26 *S. aureus* strains were isolated from anterior nasal swabs from patients with COVID-19 (SARS-CoV-2 positive test). Each strain was isolated from a different patient. The nasal swabs were collected during the screening process when the patient was admitted to the hospital in Kościerzyna (Poland). The mean age of patients was 68.9 years, where 65.4% were male (a mean age of 66.4 years) and 34.6% were female (a mean age of 73.8 years). The main clinical characteristics of the patients are summarized in Table S1.

The samples were inoculated with Columbia Blood Agar (5% sheep’s blood, Graso, Starogard Gdański, Poland) and Mannitol Salt Agar (Graso, Poland) and incubated at 37 °C for 24–48 h. Identification was based on colony morphology, a positive coagulase test (Biomed, Kraków, Poland) and the colorimetric method VITEK^®^ GP (bioMerieux, Warsaw, Poland). After their identification, all *S. aureus* strains were stored at −80 °C in Tryptic Soy Broth (Graso, Starogard Gdański, Poland) with 15% glycerol (Sigma-Aldrich, Poznań, Poland). Ethical approval was granted by the Local Independent Committee for Ethics in Scientific Research at the Medical University of Gdańsk (NKBBN/525/2021).

Twenty-one *S. aureus* strains were obtained from non-COVID-19-infected patients to compare with the COVID-19 strains also included in the study. The isolates originated from the collection of the Department of Medical Microbiology at the Medical University of Gdańsk and were selected based on their source (respiratory tract infections and bloodstream infections). To exclude the clonal identity, the *S. aureus* strains included in the study represented different types of the *spa* gene and have been characterized previously [31,55,56].

### 4.2. Cell Line and Culture Conditions

The A549 human lung carcinoma epithelial cell line (ATCC, CCL-185) (ATCC, Manassas, VA, USA) was cultured in T75 bottles (Googlab Scientific, Rokocin, Poland) in a F-12K culture medium (ATCC, Manassas, VA, USA) supplemented with 10% fetal bovine serum (FBS), 100 U/mL penicillin and 100 μg/mL streptomycin (all from Merck, Darmstadt, Germany) at 37 °C in a humidified atmosphere of 95% air and 5% CO_2_ in the Thermo Scientific Forma Steri-Cycle CO_2_ Incubator (Thermo Fisher Scientific, Waltham, MA, USA) [57,58].

### 4.3. Bacterial DNA Isolation

The isolation of genetic material from the clinical *S. aureus* isolates was conducted using a Genomic Midi AX kit (serial no. 895-20) from A&A Biotechnology (Gdynia, Poland) in accordance with the manufacturer’s guidelines. The isolated DNA was stored at −20 °C.

### 4.4. Screening for the Presence of Toxin and Adhesin Genes

The presence of toxins and adhesin genes was assessed using a PCR in an Eppendorf Mastercycler EP (Eppendorf, Hamburg, Germany) in a final volume of 25 µL.

The genes responsible for the production of toxins were A and B exfoliatins (*eta*, *etb*), enterotoxins A, B, C and D (*sea*, *seb*, *sec* and *sed*), toxic shock toxin TSST-1 (*tst*) and Panton–Valentine leucocidins (*pvls*) and were detected following previously published protocols [22,59]. For the detection of fibronectin-binding protein genes A and B (*fnbA* and *fnbB*), the parameters of the cycle were as follows: 94 °C for 1 min, 30 cycles at 94 °C for 30 s, at 50 °C for 30 s, at 72 °C for 1 min and at 72 °C for 8 min.

The amplification results were read on 2% agarose gel (Sigma-Aldrich, Poznań, Poland). The size of the amplified gene fragments was compared with the position of molecular weight markers (pUC19 DNA/MspI Marker 23; MBI Fermentas, Vilnus, Lithuania). The primer sequences and the size of the resulting products are presented in Table S2.

### 4.5. The Adhesion of the S. aureus Strains to the Pulmonary Epithelial Cells

The assay was performed following a previously published protocol with slight modifications [57,60]. In brief, the bacterial adherence to epithelial cells (A549 line) was measured by a FACSLyric flow cytometer (Becton Dickinson, Franclin Lakes, NJ, USA). The A549 cells were plated into 24-well tissue culture plates (TPP, Switzerland) at a density of 1.7 × 10^5^ per well (designed experimentally to obtain a monolayer of cells) and allowed to attach for 24 h in F12-K supplemented with 10% FBS and 100 U mL^−1^ penicillin and 100 μg mL^−1^ streptomycin. The plates were incubated at 37 °C with a 5% CO_2_ incubator. Following incubation, the plates were centrifuged at 250 g for 5 min at room temperature, the medium was removed and the A549 cell cultures were washed with 0.9% NaCl.

*S. aureus* strains were cultured in brain heart infusion (BHI) broth (Merck KGaA, Darmstadt, Germany) at 37 °C. After 24 h of incubation, the 1 mL of fresh BHI broth was inoculated with 10 µL of bacterial culture and incubated for the next 24 h. Subsequently, the bacterial cells were centrifuged at 10,000 rpm for 2 min, washed with 0.9% NaCl and stained with 10 µM of carboxyfluorescein diacetate (CFDA) (Merck KGaA, Darmstadt, Germany) for 30 min at 37 °C. The initial density of the suspension was measured by a FACSLyric flow cytometer (Becton-Dickinson, Franklin Lakes, NJ, USA) as the number of detected events per 60 s of measurement. To assess the adherence properties of the bacteria, the conditions of the experiments (initial density of suspension and time of incubation) were designed to provide the highest sensitivity for the method (current study, data not presented). The suspension of fluorescently labelled bacterial cells was transferred onto the A549 cell cultures and incubated for 30 min at 37 °C. Then, the suspension was aspirated and each cell’s inoculum was measured by a FACSLyric flow cytometer (Becton-Dickinson, Franklin Lakes, NJ, USA) once again. As a negative control, an A549 cell culture well not inoculated with bacteria was used. The obtained flow cytometric data were analyzed using Flowing Software 2.5.1 (Turku Bioscience, Turku, Finland). The bacterial adherence was estimated as the difference between the initial and final number of detected events per 60 s.

### 4.6. The Penetration of S. aureus Strains into the Pulmonary Epithelial Cells

The experiment was designed based on previously published protocols, with slight modifications [61]. In brief, the A549 cells were plated into 96-well tissue culture plates (Nest Scientific Biotechnology, Wuxi, China) at a density of 6 × 10^3^ per well and allowed to attach for 24 h in F12-K supplemented with 10% FBS. After 24 h, the medium was removed and the cells were infected with 10^6^ CFU/mL of *S. aureus* strains suspended in a fresh F12-K medium. After 6 h of incubation, a mixture of gentamycin (50 µg/mL) and streptomycin (25 µg/mL) in a 1:1 ratio was added to the medium to inactivate the bacterial cells on the outside of the pulmonary cells. After 30 min of incubation, the cells were washed three times with PBS. The cells were lysed using the following lysis buffer: 2% DOCNa (Sigma-Aldrich), 10 mM Tris and 2 mM EDTA, with a pH of 8.0 [62]. An amount of 50 µL of the suspension was removed and serial dilutions were made in 0.9% NaCl. A total of 50 µL of each dilution was spread onto LB-agar plates. The samples were then incubated overnight at 37 °C, followed by the colonies for CFU/mL counting [63].

### 4.7. The Neutral Red Viability Assay

The assay was performed as per the protocol previously published [64,65]. In brief, the A549 cells were plated into 96-well tissue culture plates (Nest Scientific Biotechnology, Wuxi, China) at a density of 6 × 10^3^ per well and allowed to attach for 24 h in F-12K medium supplemented with 10% FBS. After 24 h, the medium was removed and the 10% dimethyl sulfoxide (DMSO)-treated (Sigma-Aldrich, St. Louis, MO, USA) cells were used as a positive control, while non-treated cells were considered a negative control. The cells were infected with *S. aureus* strains suspended in a F-12K medium supplemented with 10% FBS to a final CFU/mL of 10^5^. The cells were incubated for 4 h at 37 °C in a humidified atmosphere of 95% air and 5% CO_2_. Following incubation, the supernatants were discarded and replaced with 100 µL of non-supplemented F-12K containing 0.33% neutral red (Sigma-Aldrich) with a dilution ratio of 1:40. After 2 h of incubation at 37 °C, the neutral red medium was removed and the cells were washed with 100 µL of Phosphate-Buffered Saline per well. The cells were then treated with 150 µL of a solution containing 50% ethanol (Alchem, Torun, Poland), 49% distilled H_2_O and 1% acetic acid (Alchem, Torun, Poland) and incubated with shaking at 37 °C for 10 min to extract the dye into the solution. The absorbance was measured at 540 nm (SynergyH1, BioTek Instruments, Winooski, VT, USA).

### 4.8. The Lactate Dehydrogenase (LDH) Release Assay

The LDH release assay was performed using CytoTox 96^®^ (Promega, Madison, WI, USA) as per the manufacturer’s protocol. The cells were inoculated with bacteria, as described in Section 4.5, and the experiment was performed as described earlier in our work [65].

### 4.9. Statistical Analysis

Enumeration data, shown as numbers or percentages, were compared between the groups using the Fisher exact test, with the threshold of statistical significance set at *p*-value ≤ 0.05. For other analyses, all experiments were carried out in three biological replicates. The technical replicates were averaged to produce replicate means that were used for analysis. The mean values were compared using the Kruskal–Wallis test, followed by Dunn’s multiple comparison test for the values with a nonparametric distribution. To assess if there was a significant difference between time points, the Wilcoxon signed-rank test was used. Differences were considered statistically significant if *p* ≤ 0.05.

## Figures and Tables

**Table 1:** The presence of toxin and adhesin genes in the *S. aureus* strains isolated from COVID-19 and non-COVID-19 patients.

Virulence Gene *	COVID-19 Strains n = 26 (%)	Non-COVID-19 Strains n = 21 (%)
Adhesin genes		
*fnbA*	21 (80.8)	21 (100)
*fnbB*	26 (100) **	7 (33.3)
Toxin genes		
*tst*	2 (7.7)	0
*sea*	0	4 (19.0) ***
*seb*	0	1 (4.8)
*sec*	1 (3.8)	0
*sed*	0	0
*pvl*	0	1 (4.8)
*eta*	0	0
*etb*	0	0

* Enterotoxin SEA, SEB, SEC and SED genes (*sea*, *seb*, *sec* and *sed*), exfoliative toxins ETA and ETB (*eta* and *etb*), toxic shock syndrome toxin TSST-1 (*tst*), Panton–Valentine leucocidin (*pvl*) and fibronectin-binding protein A and B genes (*fnbA* and *fnbB*). The statistical analysis was performed using Fisher’s exact test. The *S. aureus* genes with a significantly higher occurrence between the COVID-19 and non-COVID-19 strains are marked ** *p* < 0.01 and *** *p* < 0.05.

## Data Availability

Raw data available per request from the authors.

## Supplementary Materials

The following supporting information can be downloaded at: https://www.mdpi.com/article/10.3390/ijms251810050/s1.
