# Supplementary material for: Staphylococcus aureus Co-Infection in COVID-19 Patients: Virulence Genes and Their Influence on Respiratory Epithelial Cells in Light of Risk of Severe Secondary Infection
Source: Int J Mol Sci. 2024 Sep 18;25(18):10050. doi: 10.3390/ijms251810050 (PMC11431965; doi:10.3390/ijms251810050)
Supplement: Supplementary file 1 [file ijms-25-10050-s001.zip › ijms-3161035-supplementary.pdf]

Supplementary Materials to

Staphylococcus aureus Co-Infection in COVID-19 Patients: Virulence Genes and their Influence on Respiratory Epithelial Cells in Light of Risk of Severe Secondary Infection

Lidia Piechowicz [1]*, Katarzyna Kosznik-Kwaśnicka [1]*, Tomasz Jarzembowski [1], Agnieszka Daca [2], Agnieszka Necel [1], Ada Bonawenturczak [3], Olesia Werbowy [3], Małgorzata Stasiłojć [4] and Anna Pałubicka [5]

[1] Department of Medical Microbiology, Faculty of Medicine, Medical University of Gdańsk, Dębowa 25, 80-204 Gdansk, Poland

[2] Department of Pathophisiology, Medical University of Gdańsk, Dębinki 7, 80-211 Gdansk, Poland

[3] Department of Microbiology, Faculty of Biology, University of Gdańsk, Wita Stwosza 59, 80-308 Gdańsk, Poland

[4] Department of Cell Biology and Immunology, Intercollegiate Faculty of Biotechnology of University of Gdańsk and Medical University of Gdańsk, Dębinki 1, 80-211 Gdańsk, Poland

[5] Specialist Hospital in Kościerzyna Sp. z o.o., Department of Laboratory and Microbiological Diagnostics, Kościerzyna, Alojzego Piechowskiego 36, 83-400 Kościerzyna, Poland

Table S1. Main characteristics of patients hospitalized for COVID-19. M - male, F – female

No.	Age (years)	Sex	Comorbidities	Death
1	89	M	-	-
2	65	M	cardiovascular disease	-
3	71	F	diabetes, cardiovascular disease	-
4	74	M	cardiovascular disease	death
5	53	M	-	-
6	85	M	cardiovascular disease	death
7	57	M	-	-
8	73	F	diabetes, cardiovascular disease	-
9	68	F	cancer	-
10	40	M	-	-
11	47	M	-	-
12	82	F	diabetes, cardiovascular disease, chronic lung dise	death
13	73	F	diabetes, cardiovascular disease	-
14	85	F	diabetes, cardiovascular disease, chronic lung dise	-
15	68	M	-	-
16	68	M	cardiovascular disease	-
17	70	F	cardiovascular disease	-
18	54	M	diabetes, cardiovascular disease	-
19	55	M	cardiovascular disease	-
20	80	M	-	-
21	61	M	diabetes, cardiovascular disease, chronic renal dis[illegible]	-
22	89	M	diabetes, cardiovascular disease, chronic lung dise chronic renal disease	-
23	68	M	chronic lung disease, immunosuppression	-
24	67	F	-	-
25	76	M	-	-
26	75	F	cardiovascular disease, chronic lung disease	-

Table S2. **Primer sequences used in this study and size of products of PCR reaction**

Primer name	Sequence of primers (5′– 3′)	Size of product (bp)
SEA1	GGT TAT CAA TGT GCG GGT GG	102
SEA2	CGG CAC TTT TTT CTC TTC GG	
SEB1	GTA TGG TGG TGT AAC TGA GC	164
SEB2	CCA AAT AGT GAC GAG TTA GG	
SEC1	AGA TGA AGT AGT TGA TGT GTA TGG	451
SEC2	CAC ACT TTT AGA ATC AAC CG	
SED1	CCA ATA ATA GGA GAA AAT AAA AG	287
SED2	ATT GGT ATT TTT TTT CGT TC	
TSST1-1	ACC CCT GTT CCC TTA TCA TC	326
TSST1-2	TTT TCA GTA TTT GTA ACG CC	
ETA-1	GCA GGT GTT GAT TTA GCA TT	93
ETA-2	AGA TGT CCC TAT TTT TGC TG	
ETB-1	ACA AGC AAA AGA ATA CAG CG	226
ETB-2	GTT TTT GGC TGC TTC TCT TG	
PVL-1	ATCATTAGGTAAAATGTCTGGACATGATCCA	433
PVL-2	GCA TCA ACT GTA TTG GAT AGC AAA AGC	
FNBpA1	AGGATTCGCACAGCGTTGAA	3054
FNBpA2	TCCATGCCTTACGACCTCTC	
FNBpB1	ACGCCTTCATAGTGTCATTGAGT	2820
FNBpB2	GTGAAAAGCAATCTTAGATACGGCA	

M 1 2 3 M 4 5 6 7

Table S3. **Pof toxin and adhesin genes in S. aureus strains isolated from COVID-19 and non-COVID-19 patients.**

Origin	Strain no.	Toxin gene*								Adhesin gene	
		sea	*seb*	*sec*	*sed*	*eta*	*etb*	*tst*	*pvl*	*fnbA*	*fnbB*
COVID-19 patients	1	-	-	-	-	-	-	-	-	+	+
	2	-	-	-	-	-	-	-	-	-	+
	3	-	-	+	-	-	-	-	-	+	+
	4	-	-	-	-	-	-	-	-	-	+
	5	-	-	-	-	-	-	-	-	+	+
	6	-	-	-	-	-	-	-	-	+	+
	7	-	-	-	-	-	-	-	-	+	+
	8	-	-	-	-	-	-	-	-	-	+
	9	-	-	-	-	-	-	+	-	+	+
	10	-	-	-	-	-	-	-	-	+	+
	11	-	-	-	-	-	-	-	-	+	+
	12	-	-	-	-	-	-	-	-	-	+
	13	-	-	-	-	-	-	-	-	+	+
	14	-	-	-	-	-	-	-	-	+	+
	15	-	-	-	-	-	-	-	-	+	+
	16	-	-	-	-	-	-	-	-	+	+

	17	-	-	-	-	-	-	-	-	-	+
	18	-	-	-	-	-	-	-	-	+	+
	19	-	-	-	-	-	-	-	-	+	+
	20	-	-	-	-	-	-	-	-	+	+
	21	-	-	-	-	-	-	-	-	+	+
	22	-	-	-	-	-	-	-	-	+	+
	23	-	-	-	-	-	-	-	-	+	+
	24	-	-	-	-	-	-	+	-	+	+
	25	-	-	-	-	-	-	-	-	+	+
	26	-	-	-	-	-	-	-	-	+	+
non- COVID-19 patients	19K	-	-	-	-	-	-	-	-	+	+
	108	-	-	-	-	-	-	-	-	+	-
	116	-	-	-	-	-	-	-	-	+	-
	118	+	-	-	-	-	-	-	-	+	-
	121	+	-	-	-	-	-	-	-	+	-
	126	+	-	-	-	-	-	-	-	+	+
	199	-	+	-	-	-	-	-	-	+	-
	200	-	-	-	-	-	-	-	-	+	-
	202	-	-	-	-	-	-	-	-	+	-
	297	-	-	-	-	-	-	-	-	+	-
	358	-	-	-	-	-	-	-	-	+	-
	371	-	-	-	-	-	-	-	-	+	-
	7K	-	-	-	-	-	-	-	-	+	+
	10K	-	-	-	-	-	-	-	-	+	+
	12K	-	-	-	-	-	-	-	+	+	-
	104	-	-	-	-	-	-	-	-	+	-
	107	-	-	-	-	-	-	-	-	+	+
	21K	-	-	-	-	-	-	-	-	+	-
	28K	-	-	-	-	-	-	-	-	+	+
	31	+	-	-	-	-	-	-	-	+	-
	58K	-	-	-	-	-	-	-	-	+	+

*Enterotoxins SEA, SEB, SEC, SED genes (*sea, seb, sec and sed*), exfoliative toxins ETA, ETB (*eta, etb*), toxic shock syndrome toxin TSST-1 (*tst*), Panton-Valentine – leucocidin (*pvl*) and fibronectin-binging proteins A and B genes (*fnbA and fnbB*);
